# Association of ADAMTS4 and ADAMTS5 polymorphisms with musculoskeletal degenerative diseases: a systematic review and meta-analysis

**DOI:** 10.1042/BSR20181619

**Published:** 2018-11-30

**Authors:** Jian-Zhong Huo, Xing-Hua Ji, Zhong-Yi Su, Peng Shang, Fei Gao

**Affiliations:** 1 Department of Orthopedics, Shanxi Dayi Hospital, Taiyuan, Shanxi 030032, China; 2 Department of Endocrinology, the First Hospital of Shanxi Medical University, Taiyuan, Shanxi 030001, China

**Keywords:** ADAMTS4, ADAMTS5, lumbar disc degeneration, meta analysis, osteoarthritis, single nucleotide polymorphisms

## Abstract

Objective: This meta-analysis and systematic review was performed with the aim of investigating the association between a disintegrin and metalloproteinase with thrombospondin motif (ADAMTS)4, AMDMTS5 polymorphisms and risk of musculoskeletal degenerative diseases.

Methods: PubMed, EMBASE, ISI Web of Science, Wanfang and CNKI were searched from their inception until May 2018 to identify eligible studies. Data from individual studies were extracted using a standardized data collection sheet. The estimate of association between ADAMTS4, AMDMTS5 polymorphisms and risk of musculoskeletal degenerative diseases was expressed as odds ratio (OR) along with its related 95% confidence interval (95%CI) under an allelic model of inheritance. Meta-analysis was conducted using RevMan 5.3 software. Subgroup-analyses by ethnicity and type of diseases were performed.

Results: Eight studies including ten cohorts were included in this meta-analysis. The meta-analyses results based on seven studies showed that rs226794 in ADAMTS5 gene was not associated with risk of musculoskeletal degenerative diseases (A vs. G: OR 1.07; 95%CI 0.97–1.19; *P*=0.16). Rs2830585 in ADAMTS5 was significantly associated with musculoskeletal degenerative diseases in only Asians (OR 1.41, 95%CI 1.18–1.68; *P*=0.0001), but not in Caucasians. Since only two of the collected studies referred to ADAMTS4, we did not perform meta-analysis for these comparisons.

Conclusion: Taken together, rs226794 and rs2830585 in ADAMTS5 gene were not associated with musculoskeletal degenerative diseases in overall population, but there seemed to be an ethnicity-dependent effect of rs2830585 in the risk of musculoskeletal degenerative diseases. Insufficient evidence was found to support the association of other single nucleotide polymorphisms and musculoskeletal degenerative diseases.

## Introduction

Musculoskeletal degenerative diseases are a diverse group of pathological conditions affecting bone, cartilage, joint and other connective tissues. They are typically characterized by severe chronic pain, stiffness, activity limitations and incapacity in patients’ later life [1,2]. The incidence and prevalence of musculoskeletal degenerative diseases increase markedly with age, especially with the prolongation of human being lifespan over recent years [3–5]. Musculoskeletal degenerative diseases represent a great challenge to both patients and clinicians, and are becoming a serious public-health issue [6,7].

Osteoarthritis (OA) and intervertebral disc degeneration are two most common musculoskeletal degenerative diseases that share comparative similarities. The surfaces of joints are overlaid by a layer of articular cartilage covering the subchondral bone. Progressive damage of articular cartilage as with subchondral bone is the pivotal hallmark of musculoskeletal degenerative diseases. Articular cartilage consists of plenty of extracellular matrix (ECM) components that include macromolecules, collagen fibrils and large proteoglycanaggrecans [8]. Quite a small number of chondrocytes are embedded in ECM [9]. The chondrocytes can synthetize and catabolize ECM macromolecules, which correspondingly maintain the homeostasis of the extracellular microenvironment and the configuration of cartilage [9].

A disintegrin and metalloproteinase with thrombospondin motifs (ADAMTSs) in mammalian genomes contain a family of 19 secreted metalloproteinases [10,11]. All ADAMTSs belong to matrix-degrading enzymes. Regulating the structure and function of the ECM is the foremost action of the biology of ADAMTSs [11]. ADAMTS4 and ADAMTS5, also known as aggrecanase-1 and aggrecanase-2, are two members of ADAMTSs family and have been shown to degrade the cartilage proteoglycanaggrecan [12,13]. A series of studies have suggested that variants of genes encoding ECM degrading enzymes were related with OA, such as matrix metalloproteinase (MMP)-1 [14–18], MMP-3 [14,19,20] and MMP-9 [17]. Strong associations between MMP-3, MMP-9 polymorphisms and risk of lumbar disc degeneration (LDD) were also indicated [21,22]. As the earliest discovered and most efficient aggrecanases, ADAMTS4 and ADAMTS5 have received high attention in the development of musculoskeletal degenerative diseases [23].

A fully understanding of the risk factors, etiology and mechanism is significant to prevent and treat a disease. But the precise etiopathogenesis underlying musculoskeletal degenerative diseases remains unclear. Risk factors like genetics, age and joint biomechanics seem to all together result in triggering the diseases [24,25]. Mounting evidence has suggested that genetic factors play a critical role in the pathogenesis of musculoskeletal degenerative diseases [26]. So far, several studies have reported the relationship between ADAMTSs single nucleotide polymorphisms (SNPs) and musculoskeletal degenerative diseases, which have yielded inconsistent results. Therefore, we systematically reviewed the current body of evidence to summarize the association between ADAMTS4, ADAMTS5 polymorphisms and musculoskeletal degenerative diseases.

## Methods

This meta-analysis was performed in accordance with the Preferred Reporting Items for Systematic Reviews and Meta-Analyses (PRISMA) guideline [27].

### Literature search strategy

Five online databases including PubMed, EMBASE, ISI Web of Science, Wanfang and CNKI were searched from their inception until May 2018 to identify eligible studies. Structured literature search strategy based on the combination of medical subject headings (MeSH) and free text terms was used to retrieve observational studies that analyzed the association between ADAMTSs and musculoskeletal degenerative disease, no language restriction was imposed on the literature search. The literature search was performed with the following terms: (Single Nucleotide Polymorphism or polymorphism or SNP or SNPs or ‘Polymorphism, Single Nucleotide’[Mesh]) and (‘ADAMTS4 Protein’[Mesh] or ADAMTS4 or Aggrecanase-1 or A Disintegrin And Metalloproteinase With Thrombospondin Motifs 4 Protein or ‘ADAMTS5 Protein’[Mesh] or ADAMTS5 or Aggrecanase-2 or A Disintegrin And Metalloproteinase With Thrombospondin Motifs 5 Protein). A combination of related Chinese characters was used for literature search in Chinese academic databases: ‘ADAMTS’ and ‘Duo Tai Xing’. Reference sections of the acquired studies were manually searched for additional eligible studies.

### Inclusion criteria and exclusion criteria

We followed the PICOS principle to establish the eligibility criteria for the current meta-analysis. Studies satisfying the following criteria were included for review: (1) patients to be included should be diagnosed as musculoskeletal degenerative diseases, such as OA and LDD; (2) published or unpublished studies on the relationship between ADAMTS4 or ADAMTS5 polymorphisms and musculoskeletal degenerative diseases susceptibility; (3) control subjects should be defined as healthy subjects without history of musculoskeletal degenerative diseases; (4) available odds ratio (OR) and 95%CI under allelic comparison of individual loci; (5) observational studies (case–control or cohort studies) on humans.

Correspondingly, studies would be excluded if they met the following exclusion criteria: (1) animal studies, reviews, case reports, conference abstracts, as well as comments and editorials; (2) data that overlapped previous publications. If duplicated studies reporting overlapping data were identified, the most comprehensive one was included in the meta-analysis.

### Data extraction

Two independent reviewers extracted detailed information according to a pre-specified data collection sheet, data extracted included: (1) surname of the first author; (2) year of publication; (3) variant locus; (4) country where the study was performed; (5) ethnicity of enrolled subjects; (6) diagnosis of cases; (7) sample size; (8) results of Hardy–Weinberg equilibrium (HWE) test; (9) major allele and minor allele distribution of case and control participants; (10) *P* value for the associations. Once encountering discrepancy during this process, two investigators re-inspected the article together and reached an agreement through discussion.

### Quality assessment

The Newcastle–Ottawa Scale (NOS) for the assessment of non-randomized studies was used to assess the methodological quality of case–control studies and cohort studies [28]. Three broad perspectives including selection of cases and controls, comparability of the groups and ascertainment of outcome of interest were assessed using the Star system (http://www.ohri.ca/programs/clinical_epidemiology/oxford.asp). Higher score indicates higher methodological quality and the maximum score is 9 Stars. Two reviewers (J.H. and X.J.) independently assessed the methodological quality of included studies. The results of risk of bias assessment were compared afterwards. In case of discrepancies between two reviewers, the third reviewer (F.G.) was consulted.

### Statistical analysis

The strength of association between ADAMTS4, ADAMTS5 polymorphisms and musculoskeletal degenerative diseases susceptibility was assessed through pooling ORs and the corresponding 95%CI of individual studies. The between-study heterogeneity was assessed using the *Q*-statistical test and *I*^2^ test, where *P*>0.1 and *I*^2^ < 50% indicate acceptable heterogeneity [29]. In our meta-analysis, patients diagnosed with different types of musculoskeletal degenerative diseases could be included. Therefore, we combined the OR of each study using a random-effect model regardless of the heterogeneity detected between included studies because random-effect model accommodates diversity between studies and is preferable in the presence or anticipation of interstudy variances [30,31].

The leave-one-out sensitivity analysis was performed by omitting each study in turn and re-evaluating the resulting effect on the overall effect. Egger’s regression test and Begg’s rank correlation test were used to estimate the publication bias (Stata version 12.0, Stata Corp LP, U.S.A.) [32]. Forest plots and funnel plots were generated using RevMan 5.3 software (Copenhagen: The Nordic Cochrane Centre, The Cochrane Collaboration, 2014). In addition, stratified analyses were performed according to subtypes of musculoskeletal degenerative diseases and ethnicity of cases.

### Functional predictions

In order to perform functional annotation of rs2830585 and rs26932893, we applied an *in silico* analysis using HaploReg 4.1 database (http://pubs.broadinstitute.org/mammals/haploreg/haploreg.php) [33]. The Genotype-Tissue Expression (GTEx) project is a combination of resource database and associated tissue bank to study the relationship between genetic variation and gene expression in human tissues (https://gtexportal.org/home/) [34]. GTEx was used to test if rs2830585 and rs26932893 in ADAMTS5 gene were associated with altered expression of ADAMTS5 in human tissues. Additionally, PROMO (http://alggen.lsi.upc.es/cgi-bin/promo_v3/promo/promoinit.cgi?dirDB=TF_8.3) was used to identify putative transcription factor binding sites in rs2830585 and rs26932893 [35].

## Results

### Study selection

**Figure 1:**
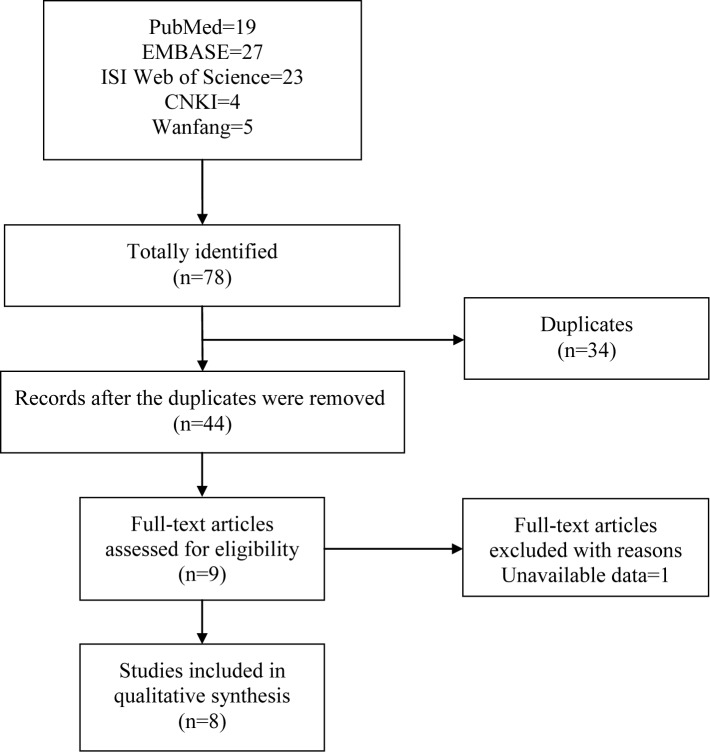
Flow diagram of literature search and screen

Combining the search results of all databases, a total of 78 potentially relevant articles were retrieved with 19 from PubMed, 27 from EMBASE, 23 from ISI Web of Science, 5 from Wanfang and 4 from CNKI. No additional articles were obtained through the manual search. After removing 34 records of duplicates, 44 articles remained for further screen. Another 35 articles were eliminated after screening for eligibility with titles and abstracts. Only 1 article was removed after evaluating the full text because of unavailable data [36]. Finally, 8 articles were deemed eligible and were subsequently incorporated into this systematic review [37–44]. The literature selection process was presented in Figure 1.

### Study characteristics

All included studies were based on a case–control design. Seven studies [37,39–44] were performed among populations of Asian ancestry but Rodriguezs’ [38] was among Caucasian ancestry with four different cohorts. Four of the included studies were about OA, and the others were about LDD. The OA was diagnosed according to the American College of Rheumatology criteria, and radiographic featured were assessed using the Kellgren–Lawrence score system. The diagnosis of LDD was made based upon the confirmation of MRI scanning. There were seven studies [37–39,41–43] looking at ADAMTS5, one looking at only ADAMTS4 [40], and one focusing on both of them [44]. The rs2830585 and rs226794 SNPs in ADAMTS5 were the mostly frequently investigated polymorphisms among these studies, consequently we combined the ORs from each individual study using an allele genetic model. The main characteristics of the 8 included studies were summarized in Tables 1 and 2. According to the NOS, our included studies gained an average of 6 Stars, the risk of bias for the included studies was summarized in Table 3.

**Table 1:** Main characteristics of included studies on ADAMTS5 polymorphisms

Study on ADAMTS5	Variant	Country	Ethnicity	Sample size	Diagnosis	Major allele (*N*)		Minor allele (*N*)		*P*-value for association	HWE
						Case	Control	Case	Control		
Canbek, 2016 [44]	*rs226794 (G/A)*	Turkey	Caucasian	95/80	OA	163	139	27	21	0.178	0.28
	*rs2830585(C/T)*	Turkey	Caucasian	95/80	OA	159	141	31	19	0.81	0.65
Chen, 2015 [43]	*rs226794 (G/A)*	China	Han	166/204	OA	163	188	169	220	>0.05	0.81
	*rs1974415 (A/G)*	China	Han	166/204	OA	208	247	124	161	>0.05	0.87
	*rs2132824 (G/A)*	China	Han	166/204	OA	221	297	107	111	>0.05	0.76
	*rs151065 (A/G)*	China	Han	166/204	OA	165	225	167	183	>0.05	0.99
Gu, 2013 [42]	*rs226794 (G/A)*	China	Han	420/312	OA	268	182	572	442	0.261	0.46
	*rs2830585(C/T)*	China	Han	420/312	OA	446	276	394	348	**0.001**	**<0.01**
Jiang, 2017 [41]	*rs162509 (C/T)*	China	Han	828/400	LDD	288	330	568	470	**0.001**	0.41
Rajasekaran, 2015 [39]	*rs162509 (C/T)*	India	India	387/308	LDD	NR	NR	NR	NR	**0.04068**	NR
Rodriguez, 2008 [38]	*rs226794 (G/A)*	Spain	Caucasian	826/294	OA	1491	524	161	64	NR	>0.05
	*rs2830585(C/T)*	Spain	Caucasian	826/294	OA	1299	479	353	109	NR	>0.05
Fytili, 2005 [38]	*rs226794 (G/A)*	Greece	Caucasian	159/193	OA	293	348	25	38	0.4	>0.05
Mustafa, 2005 [38]	*rs226794 (G/A)*	U.K.	Caucasian	1465/698	OA	2630	1241	300	155	NR	>0.05
Wu, 2014 [37]	*rs226794 (G/A)*	China	Han	489/558	LDD	438	459	492	503	0.7884	0.96
	*rs2830585(C/T)*	China	Han	489/558	LDD	846	917	66	97	0.0667	0.95
	*rs162509 (C/T)*	China	Han	489/558	LDD	385	411	525	603	0.4299	0.95
	*rs1974415 (A/G)*	China	Han	489/558	LDD	579	591	351	373	0.6703	0.32
	*rs2132824 (G/A)*	China	Han	489/558	LDD	676	693	254	269	0.7517	0.61
	*rs151065 (A/G)*	China	Han	489/558	LDD	486	546	426	468	0.8068	0.98

Abbreviations: HWE, Hardy–Weinberg equilibrium; LDD, lumbar disc degeneration; NR, not reported; OA, osteoarthritis.

Statistically significant (*P*<0.05) findings were highlighted in bold.

Fytili, 2005 and Mustafa, 2005 are two cohorts in the Rodriguez, 2008 study, so we used Rodriguez, Fytili and Mustafa to represent different cohorts.

**Table 2:** Main characteristics of included studies on ADAMTS4 polymorphisms

Study ADAMTS4	Variant	Country	Ethnicity	Sample size	Diagnosis	Major allele (*N*)		Minor allele (*N*)		*P*-value for association	HWE
						Case	Control	Case	Control		
Canbek, 2016 [44]	*rs4233367 (C/T)*	China	Han	95/80	OA	132	105	58	55	0.712	0.96
Liu, 2016 [40]	*rs4233367 (C/T)*	China	Han	482/496	LDD	889	880	75	108	NR	0.36
	*rs11585858 (A/C)*	China	Han	482/496	LDD	308	312	656	660	NR	0.98
	*rs41270041 (C/G)*	China	Han	482/496	LDD	44	45	918	929	NR	0.61
	*rs10908826 (C/T)*	China	Han	482/496	LDD	653	666	311	326	NR	0.42

Abbreviations: HWE, Hardy–Weinberg equilibrium; LDD, lumbar disc degeneration; NR, not reported; OA, osteoarthritis.

Statistically significant (*P*<0.05) findings were highlighted in bold.

**Table 3:** Quality assessment of included studies

Item/Study	Canbek, 2016	Chen, 2015	Gu, 2013	Jiang, 2017	Liu, 2016	Rajasekara, 2015	Rodriguez, 2008	Wu, 2014
Adequate definition of cases	*	*	*	*	*	*	*	*
Representativeness of cases	–	–	*	–	–	–	–	–
Selection of control subjects	–	–	–	–	–	–	–	–
Definition of control subjects	*	*	*	*	*	*	*	*
Control for important factor or additional factor	–	*	*	*	**	–	–	**
Exposure assessment	*	*	*	*	*	*	*	*
Same method of ascertainment for all subjects	*	*	*	*	*	*	*	*
Non-response rate	*	*	*	*	*	*	*	*

A study could be awarded a maximum of one star for each item except for the item ‘Control for important factor or additional factor’.

The definition/explanation of each column of the Newcastle–Ottawa Scale is available from (http://www.ohri.ca/programs/clinical_epidemiology/oxford.asp).

### Association between ADAMTS5 and musculoskeletal degenerative diseases

#### Rs226794, rs2830585 and musculoskeletal degenerative diseases

**Figure 2:**
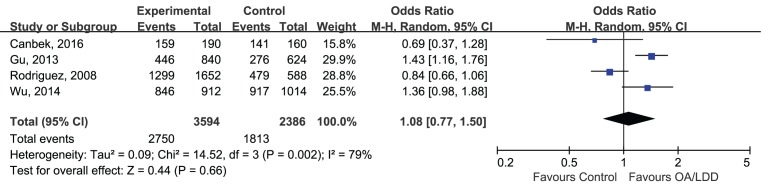
Forest plot of rs2830585 in ADAMTS5 gene and risk of musculoskeletal degenerative diseases using a random effect model

**Figure 3:**
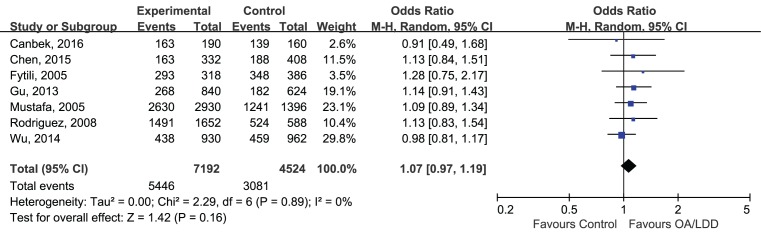
Forest plot of rs26794 in ADAMTS5 gene and risk of musculoskeletal degenerative diseases using a random effect model

For the reason that most of the included studies are lacking of available genotypes, we just analyze the allelic comparison of rs2830585 and rs226794, because the allele distributions in cases and controls could provide useful, direct summary statistics for the data. The data were analyzed by Chi-squared and *I*^2^ to detect the between-study heterogeneity. In this meta-analysis, the effect size of each included study was combined using random-effect model because there was an anticipated heterogeneity across study. The merged ORs suggested that for both rs2830585 and rs226794 polymorphisms, no pooled correlations with musculoskeletal degenerative diseases were identified under the allelic model when analyzing all of the relevant studies together. For rs2830585, four studies were pooled (C vs. T: OR 1.14; 95%CI 0.99–1.30; *P*=0.06. Figure 2), the result suggested no association between rs2830585 and musculoskeletal degenerative diseases. But there was a significant level of heterogeneity detected (*P*=0.002,

= 79%). For rs226794, summarized data was based on seven studies (A vs. G: OR 1.07; 95%CI 0.97–1.19; *P*=0.16, Figure 3) with a non-significant level of heterogeneity (*P*=0.89, *I*^2^ = 0).

To test if there was an ethnicity-specific effect on these associations, subgroup analysis by ethnicity was performed for both polymorphisms (Table 4). For rs2830585 and musculoskeletal degenerative diseases, negative results were found in both Asian and Caucasian. However, we observed an ethnicity-dependent effect in rs2830585, rs2830585 was not associated with risk of musculoskeletal degenerative diseases in the overall population, but it was significantly associated with musculoskeletal degenerative diseases in Asian (OR 1.41, 95%CI 1.18–1.68; *P*=0.0001), this finding was further strengthened by low heterogeneity between studies among Asian populations (*P*=0.80, *I*^2^ = 0%). We also performed subgroup analysis according to the diagnosis of patients (OA or LDD), studies focusing on different kinds of musculoskeletal degenerative diseases were combined in different subgroups. Based on the results of subgroup analysis, rs226794 was not associated with risk of OA or LDD, neither was rs2830585 (Table 4).

**Table 4:** Subgroup analysis of rs226794, rs2830585 and risk of musculoskeletal degenerative diseases

Variant	Subgroup	Category	No. of studies	OR (95%CI)	*P*-value	Test for heterogeneity
Rs226794	Ethnicity	Asian	3	1.05 (0.93, 1.20)	0.42	*P*=0.51, *I*^2^ = 0%
		Caucasian	4	1.11 (0.95, 1.29)	0.21	*P*=0.87, *I*^2^ = 0%
	Type of disease	OA	6	1.12 (0.99, 1.26)	0.06	*P*=0.98, *I*^2^ = 0%
		LDD	1	0.98 (0.81, 1.17)	0.79	NA
Rs2830585	Ethnicity	Asian	2	**1.41 (1.18, 1.68)**	**0.0001**	*P*=0.80, *I*^2^ = 0%
		Caucasian	2	0.82 (0.65, 1.02)	0.07	NA
	Type of disease	OA	3	1.10 (0.94, 1.27)	0.23	***P*=0.001, *I*^2^ = 85%**
		LDD	1	1.36 (0.98, 1.88)	0.07	NA

95%CI: 95% confidence interval; LDD: lumbar disc degeneration; NA: not available; OA: osteoarthritis; OR: odds ratio.

Statistically significant results were highlighted in bold.

**Figure 4:**
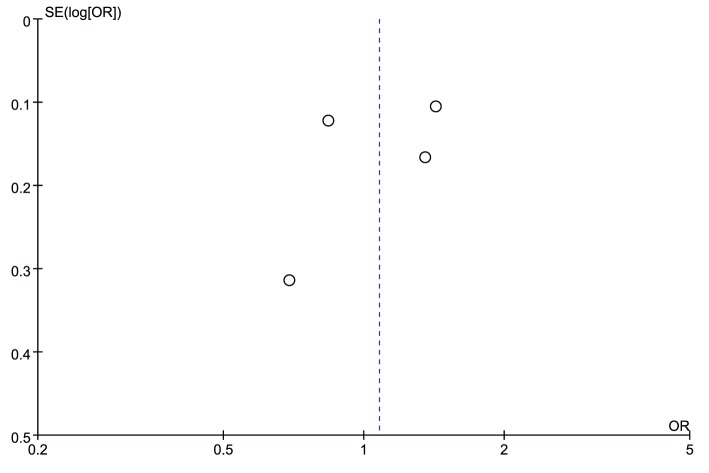
Funnel plot of rs2830585 in ADAMTS5 gene and risk of musculoskeletal degenerative diseases

**Figure 5:**
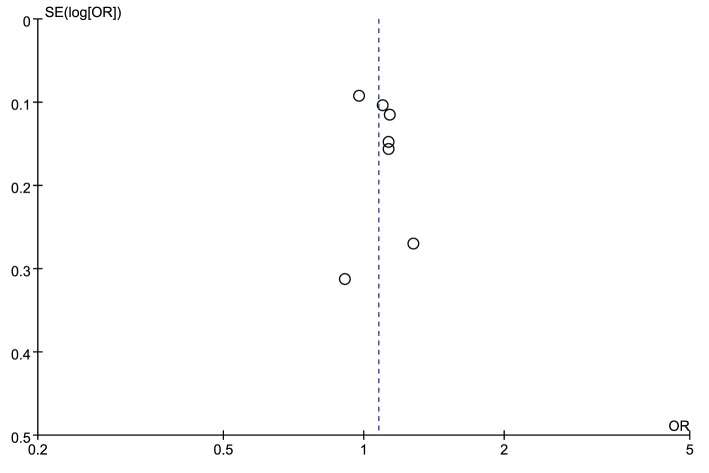
Funnel plot of rs226794 in ADAMTS5 gene and risk of musculoskeletal degenerative diseases

The leave-one-out sensitivity analysis was carried out to evaluate the stability of the overall effect for both rs2830585 and rs226794, and the results confirmed the non-significant association between rs2830585, rs226794 and musculoskeletal degenerative diseases (detailed data not shown). The funnel plots for rs2830585 (Figure 4) and rs226794 (Figure 5) were both visually symmetrical, the results of Begg’s test (rs2830585: *z* = 0.60, *P*=0.548; rs226794: *z* = 0.34, *P*=0.734) and Egger’s test (rs2830585: *t* = 0.82, *P*=0.448; rs226794: *t* = −0.57, *P*=0.628) also indicated no statistically significant publication bias.

#### Other polymorphisms of ADAMTS5 and musculoskeletal degenerative diseases

Since only few studies reported detailed data about the association between rs1974415, rs2132824, rs151065, rs162509 and risk of musculoskeletal degenerative diseases, these results were not combined using a meta-analysis, providing a narrative description as an alternative. Detailed data about these polymorphisms and risk of musculoskeletal degenerative diseases were summarized in Table 1.

Chen et al. [43] investigated the correlation of several SNPs about ADAMTS5 and OA among Chinese population. A linkage disequilibrium in SNPs of rsl51065-rs229077-rs56153501-rs2830585-rs58215296 was detected, and the haplotype of G-T-A-C-A of the SNPs was proportional to the susceptibility of OA (OR 1.874, 95%CI 1.019–3.446; *P*<0.05).

Jiang et al. [41] looked at rs162509 and lumbar spine pathologies through Chinese population. The G allele frequency of the case subjects was significantly higher than the control subjects (OR 1.38; 95%CI 1.13–1.69; *P*=0.001).

In Rajasekaran et al.’s study [39], rs162509 of ADAMTS5 were found to be significantly linked to the severity of disc degeneration (OR 1.281, *P*=0.04068). The study led by Gu et al. [42] looked at rs2380585 C/T and rs226794 G/A and OA occurred in cervical, lumber, knee and hand among Chinese population. In terms of rs226794, insignificantly statistical difference existed between OA and control participants under allelic and genotypic frequency comparison (*P*=0.261). With regard to rs2830585, T allele carrier seemed to have a lower risk for the cervical OA (OR 0.664, 95%CI 0.521, 0.847; *P*=0.001) and overall OA vulnerability (OR 0.701, 95%CI 0.569, 0.863; *P*=0.001).

Rodriguez et al.’s study [38] was conducted among European population from three countries (U.K., Spain and Greece). The initial study suggested that T allele of rs226794 might play a protective role towards OA susceptibility, while the initial finding was not replicated by additional studies. None of the remaining SNPs or haplotypes constructed with these SNPs presented to be significantly associated with OA susceptibility.

### Association between ADAMTS4 polymorphisms and musculoskeletal degenerative diseases

Since only two of the collected studies referred to ADAMTS4, we did not perform meta-analysis for these comparisons. Liu et al. [40] genotyped five tag SNPs in and around ADAMTS4 gene, and four of them (rs11585858, rs41270041, rs4233367 and rs10908826) were further studied. Only rs4233367 presented significant difference under both allelic and genotypic comparisons between case and control subjects. The T allele was found to have a declined susceptibility to LDD (OR 0.69, 95%CI 0.50, 0.94; *P*=0.0166). Subjects with TT genotype also had significantly lower risk of LLD than subjects with CC genotype (OR 0.21, 95%CI 0.0461, 0.99; *P*=0.0374). Canbek et al.’s study [44] investigated rs226794 and rs2830585. For them, neither genotype nor allele distribution was observed to have an association with KOA. In addition, Canbek et al. [44] conducted stratification analysis based on gender, but still failed to find any positive association between KOA vulnerability and these SNPs.

### Functional predictions

**Figure 6:**
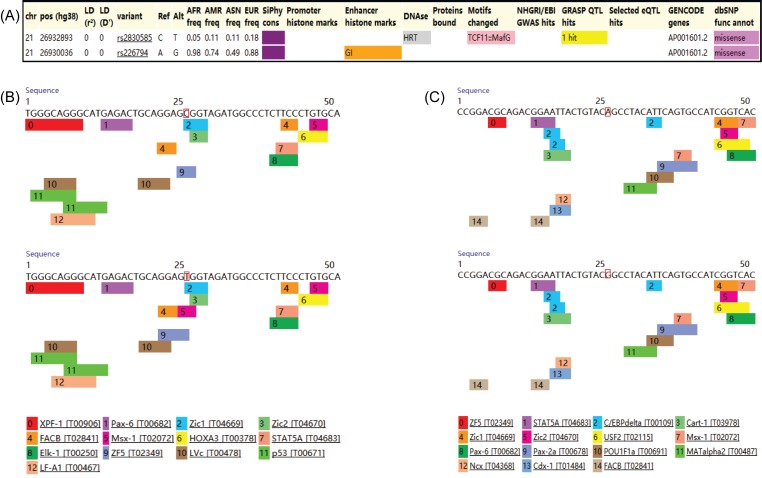
Functional predictions of rs2830585 and rs226794 (**A**) HaploReg view of rs2830585 and rs26794; (**B**) predicted transcription factor binding sites in rs2830585; (**C**) predicted transcription factor binding sites in rs226794.

In the GTEx database, no evidence was found to support the association between rs2830585, rs226794 and regulation of ADAMTS5 expression in any kind of human tissue (detailed data not shown). The effect of SNPs on expression from eQTL studies from HaploReg suggested that only rs2830585 was associated with altered expression of ADAMTS5 in human glioblastoma cell line U87MG, no promoter histone marks and enhancer histone marks were found in these variants (Figure 6A). According to the results of PROMO search, the predicted transcription factor binding sites remained unchanged in both polymorphisms (Figure 6B,C).

## Discussion

Despite extensively studied, the etiology and pathogenesis of musculoskeletal disorders have not been entirely understood. A large body of studies has indicated that genetic susceptibility in collaboration with environmental effects contributes to the etiopathogenesis of musculoskeletal degenerative diseases [11,45,46]. In fact, according to some reports, genetic factors appear to be predominantly responsible for the development of musculoskeletal degenerative diseases such as OA and LDD, with environment playing a much less significant role [47–49]. The identification of variants conferring susceptibility to musculoskeletal degenerative diseases could help to develop individualized predictive model, and thus to anticipate the phenotype of musculoskeletal degenerative diseases in the future [50].

ADAMTS genes encode a series of secreted ADAMTS proteases which were known to regulate development, angiogenesis, coagulation, as well as homeostasis in the ECM [11]. Current studies have shown that variants within several ADAMTS genes are associated with musculoskeletal degenerative diseases like OA [51–53], LDD [39] and Achilles tendon pathology [54]. Several ADAMTSs have been discovered to be expressed in connective tissues [55]. Nevertheless, functional explorations of ADAMTSs have largely been limited to only a few specific members, exceptionally ADAMTS4 and ADAMTS5, which have been involved in the occurrence and progression of cartilage damage [56]. The remaining members are not prone to function since their low activity or even absence in the tissues, these members are not likely to play a critical role in musculoskeletal degenerative diseases [13]. Therefore, in our current study, we only focused on the variants within these two genes and the risk of musculoskeletal degenerative diseases.

ADAMTS4 and ADAMTS5 genes locate at chromosome 1q23 and 21q21.3, respectively, the corresponding proteases they encode share similar domain arrangement and comparable function [13]. Prasadam et al. [57] reported that irregular interplay between cartilage chondrocytes and subchondral bone osteoblasts significantly influence the critical features of both bone and cartilage by producing abnormal levels of ADAMTS4 and ADAMTS5. Both ADAMTS4 and ADAMTS5 have been recognized as biomarkers to assess the severity of early stage osteoarthritic cartilage damage in many *in vivo* and *ex vivo* experimental studies.

Rodriguez et al. [38] predicted the potential role of rs226794 and rs2830585 in musculoskeletal degenerative diseases using SIFT and PolyPhen, and they reported the potentially damaging effect of rs226794 but not that of rs2830585. However, with the application of SNPs3D they confirmed that both polymorphisms have potential deleterious effects on musculoskeletal degenerative diseases. The functional predictions of these two polymorphisms were based on screening of amino acid conservation, and the high degree of conservation of both polymorphisms is owing to their location in functional domains. Rs2830585 maps to the first of the two thrombospondin type I domain of ADAMTS5, while rs226794 maps to a functional domain of unknown function in exon 7. However, based on the findings of our present meta-analysis, rs2030585 and rs226794 were not associated with musculoskeletal degenerative diseases in the overall population. Insignificant results were also yielded in subgroup-analyses by types of diseases. The only statistically significant finding was that rs2830585 represented a risk factor for the development of musculoskeletal degenerative diseases in Asians (OR 1.14, 95%CI 1.18, 1.68; *P*=0.0001). There seemed to be an ethnicity-dependent effect in the association between rs2830585 and risk of musculoskeletal degenerative diseases, but whether the variation in ethnicity leads to different results needs to be confirmed by further studies within different ethnic backgrounds.

In order to confirm the negative findings of our present study, we performed functional predictions of rs226794 and rs2830585 using several online databases including GTEx, PROMO and HaploReg. We failed to find any evidence to support the hypotheses that rs226794 or rs2830585 could regulate the expression of ADAMTS5 in any kind of human tissues. The findings based on PROMO also suggested that predicted transcription factor binding sites remained unchanged in both polymorphisms. The HaploReg results further confirmed that no promoter or enhancer histone marks were associated with these variants. Taken together, reasonable confidence should be placed to the null findings of the association between ADAMTS5 polymorphisms and risk of degenerative musculoskeletal diseases.

During the past two decades, there has been a far-reaching transformation in the ability to distinguish genes that contribute to various diseases, so as to the number of novel genetic polymorphisms in relation to musculoskeletal degenerative diseases are surging. Unfortunately, under most circumstances, researchers have less interest to further study these previously reported SNPs. In addition, these studies indeed suffered from large heterogeneity that was caused by diverse ethnic origin of the participants, sex and age variation, as well as the difference of diagnostic criteria and so forth. Both the aforementioned factors made it impossible to perform a formal meta-analysis of these studies. In this meta-analysis and systematic review, we summarized the available literature about SNPs in ADAMTS4 and ASAMTS5 genes, thus providing an analysis of the correlation between these genetic polymorphisms and musculoskeletal degenerative diseases vulnerability. There are several genetic models that could be used in meta-analysis of association studies, such as recessive model, allele model, dominant model and co-dominant model. Although all these aforementioned models are valid methods for analysis of an association study, any such study should have a pre-specified analysis plan because applying all tests will definitely increase the probability of a false-positive result. We only synthesize the two SNPs with allelic comparison in this systematic review, because the allele distributions in cases and controls could provide useful and direct summary statistics for the data. For rs2830585 and rs226794, the merged ORs and 95%CIs did not appear to be associated with musculoskeletal degenerative diseases. However, the result could not represent the overall population because six of the eight studies were performed across Asian population. Studies with larger samples conducted in various populations are required to obtain a clear understanding of these possible associations. Another limitation shouldn’t be ignored when interpreting the results was that there are several subtypes of OA and LDD. But a meta-analysis based on OA and LDD subtype could not be conducted because of the quantity limitation of currently available literature. Therefore, more well-designed clinical materials along with functional analyses are required to exactly elucidate the mechanisms of ADAMTS polymorphisms in the pathogenesis of musculoskeletal degenerative diseases.

In summary, our study elaborated the association of ADAMTS4, ADAMTS5 polymorphisms and risk of musculoskeletal degenerative diseases. Based upon the findings of our present study, rs226794 and rs2830585 in ADAMTS5 gene were not associated with musculoskeletal degenerative diseases in overall population, but there seemed to be an ethnicity-dependent effect of rs2830585 in the risk of musculoskeletal degenerative diseases since rs2830585 conferred a significantly higher risk of musculoskeletal degenerative diseases in Asians. Insufficient evidence was found to support the association of other SNPs and musculoskeletal degenerative diseases. Considering the limitations of our meta-analysis, additional studies with larger sample-size among other ethnicities are encouraged to elucidate the real effect of these polymorphisms and musculoskeletal degenerative diseases.

## Abbreviations

ADAMTS: A disintegrin and metalloproteinase with thrombospondin motif
GTEx: Genotype-Tissue Expression
HWE: Hardy–Weinberg equilibrium
IVDD: intervertebral disc degeneration
LDD: lumbar disc degeneration
MeSH: Medical subject headings
MMP: matrix metalloproteinase
NOS: Newcastle–Ottawa Scale
NR: not reported
OA: osteoarthritis
OR: Odds ratio
SNP: single nucleotide polymorphism
